# Checking NEKs: Overcoming a Bottleneck in Human Diseases

**DOI:** 10.3390/molecules25081778

**Published:** 2020-04-13

**Authors:** Andressa Peres de Oliveira, Luidy Kazuo Issayama, Isadora Carolina Betim Pavan, Fernando Riback Silva, Talita Diniz Melo-Hanchuk, Fernando Moreira Simabuco, Jörg Kobarg

**Affiliations:** 1 Instituto de Biologia, Departamento de Bioquímica e Biologia Tecidual, Universidade Estadual de Campinas, Campinas, São Paulo 13083-862, Brazil; andressa2401@gmail.com (A.P.d.O.); kazuo.bio@gmail.com (L.K.I.); isadora.bpavan@gmail.com (I.C.B.P.); fernandoriback@hotmail.com (F.R.S.); talitadinizmelo@yahoo.com.br (T.D.M.-H.); 2 Faculdade de Ciências Farmacêuticas, Universidade Estadual de Campinas, Campinas, São Paulo 13083-871, Brazil; 3 Laboratório Multidisciplinar em Alimentos e Saúde, Faculdade de Ciências Aplicadas, Universidade Estadual de Campinas, São Paulo 13484-350, Brazil; simabuco@gmail.com

**Keywords:** NEKs, cancer, disorders

## Abstract

In previous years, several kinases, such as phosphoinositide 3-kinase (PI3K), mammalian target of rapamycin (mTOR), and extracellular-signal-regulated kinase (ERK), have been linked to important human diseases, although some kinase families remain neglected in terms of research, hiding their relevance to therapeutic approaches. Here, a review regarding the NEK family is presented, shedding light on important information related to NEKs and human diseases. NEKs are a large group of homologous kinases with related functions and structures that participate in several cellular processes such as the cell cycle, cell division, cilia formation, and the DNA damage response. The review of the literature points to the pivotal participation of NEKs in important human diseases, like different types of cancer, diabetes, ciliopathies and central nervous system related and inflammatory-related diseases. The different known regulatory molecular mechanisms specific to each NEK are also presented, relating to their involvement in different diseases. In addition, important information about NEKs remains to be elucidated and is highlighted in this review, showing the need for other studies and research regarding this kinase family. Therefore, the NEK family represents an important group of kinases with potential applications in the therapy of human diseases.

## 1. Introduction

**Figure 1:**
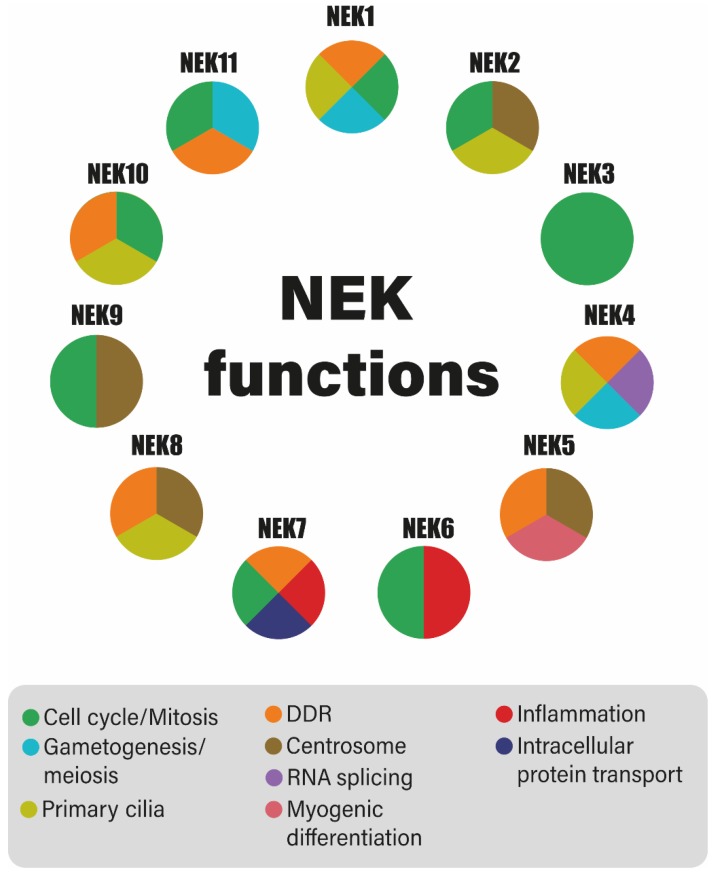
Schematic overview of the functions of NEK (Never in Mitosis A (NIMA)-related kinase) family members. In the clockwise direction the pie charts represent the described functions for each member of the NEK family, as shown in the subtitles at the bottom. DDR: DNA Damage Response.

In humans, the Never in Mitosis A (NIMA)-related kinases, or simply NEKs, belong to a family with eleven different members that share significant homology with *Aspergillus nidulans* NIMA proteins [1,2]. Differences, especially in the C-terminal regulatory and docking domains of each family member, suggest that they have distinct functions [3]. NEKs were initially characterized by their function in regulating mitosis, being the least characterized family of kinases involved in this process, aside from the cyclin-dependent kinase (CDK), and Polo and Aurora kinase families [4,5,6]. However in recent years, new functions have been designated to the NEK family members, especially those related to primary ciliary and DNA damage response functions [7]. Members of the NEK family participate in one or more of the functions shown in Figure 1. Cell cycle: NEK1 [8], NEK2 [9], NEK3 [10], NEK6 [11], NEK7 [12], and NEK10 [13]; mitosis: NEK6 [14], NEK7 [15], NEK8 [7], and NEK9 [16]; meiosis: NEK1 [17], NEK4 [18], and NEK11 [19]; centrosome organization: NEK2 [20], NEK5 [21], NEK8 [7], and NEK9 [16]; gametogenesis: NEK1 [22]; primary cilia: NEK1 [23], NEK2 [24], NEK4 [25], NEK8 [7], and NEK10 [26]; RNA Splicing: NEK4 [18]; myogenic differentiation: NEK5 [27]; inflammation: NEK6 [28], and NEK7 [29]; intracellular protein transport: NEK7 [12]; and finally, the DNA damage response (DDR): NEK1 [30], NEK4 [31], NEK5 [32], NEK7 [12], NEK8 [33], NEK10 [13], and NEK11 [34]. Cell cycle deregulation, defects in ciliary functions, and the response to DNA damage are known characteristics of several diseases, such as the development of tumors, which is one of the most characterized and studied areas [35,36].

**Figure 2:**
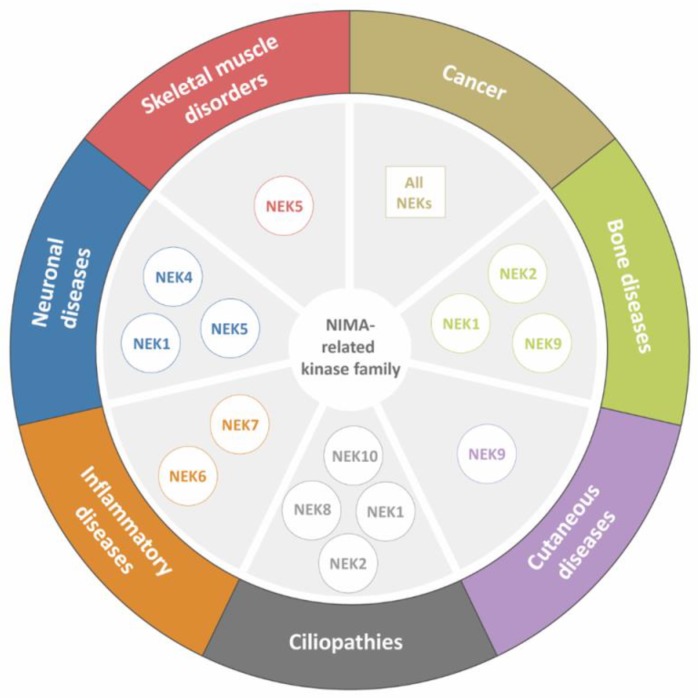
The NIMA-related kinase (NEK) family is involved in various human diseases. The schematic circle shows that the 11 members of the NEK family are related to different human diseases, such as cancer, bone, neuronal, skeletal muscle, inflammatory, cutaneous, and ciliopathic diseases. All NEKs are involved in cancer.

**Figure 3:**
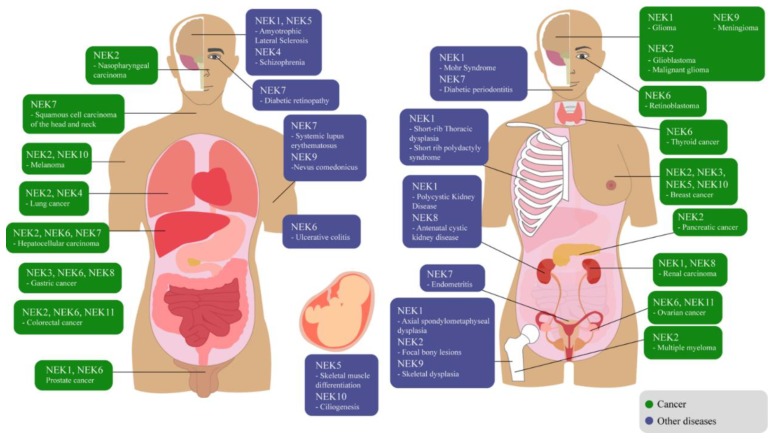
Schematic overview of NEKs and human diseases. At the top left, a human embryo is represented. It shows the relevance of NEK5 and NEK10 in the development of some tissues. In the middle, the correlations between NEKs and human diseases are presented, for each corresponding organ. There are no major differences between men and women, except for the reproductive organs and breasts. For schematic purposes, the figure only highlights some organs for each sex.

Members of the NEK family are involved in the development and/or progression of several diseases [4,7,35,36]. In recent years, information about the NEK family has been accumulating [7,37,38,39], although there is a large gap in knowledge for some lesser-studied members of the family. Thus, considering the main functions of the NEK family, this review outlines the individual roles of each family member and their involvement in diverse types of diseases, as shown in Figure 2. Figure 3 summarizes the diseases described in this review and indicates the organ or tissue affected.

## 2. NEK1

NEK1 is a widely studied kinase from the NEK family, and its key role in several cellular functions is still a focus of human disease studies, since alterations in its expressions can lead to several pathologies. It was demonstrated to have pleiotropic effects and a correlation with Polycystic Kidney Disease (PKD) through underexpression in mice, which also suggested a ciliary function [23]; this was confirmed by subsequent studies [8]. Additionally, NEK1 is involved in cell cycle progression and many DNA Damage Response (DDR) pathways. NEK1 was shown to interact with several DNA repair proteins, suggesting that it plays a key role in the DNA damage response (DDR) [30]. Finally, it has been suggested that NEK1 is involved in gametogenesis, due to its high expression levels in meiotic tissues [17] and important roles in spermatogenesis [22] and spindle assembly in meiosis I [17]. Thus, considering the versatility of NEK1, further research on its role in human disorders and treatments should be conducted.

Amyotrophic Lateral Sclerosis (ALS) is a neurodegenerative disorder that causes the death of motoneurons (MN) responsible for controlling voluntary muscle activity, leading to muscle denervation and, consequently, gradual loss of motor functions such as moving and speaking, and, as a consequence, eventual death by respiratory failure. In the last decade, many reports have suggested that several genes are related to familial ALS (FALS) and sporadic ALS (SALS), including superoxide dismutase 1, fused in sarcoma (FUS), hexanucleotide repeat expansion in C9ORF72, Ataxin-2, Optineurin, Valosin-containing protein, Profilin 1, Ubiquilin 2 and Ubiquilin 4, Matrin 3, Coiled-coil-helix-coiled-coil-helix domain containing 10, senataxin, TANK binding kinase 1, kinesin heavy chain isoform 5A, and NEK1, as described next. Cirulli et al. (2015) [40] first suggested the association between NEK1 and ALS, reporting a higher heterozygous loss-of-function mutation profile of NEK1 in ALS patients compared with an asymptomatic control group. Subsequently, several studies corroborated this initial speculation. Following the first whole-exome analysis, Kenna et al. [41] and Brenner et al. [42] demonstrated variants of NEK1 and susceptibility to familial ALS. An analysis of the whole-exome sequence data from familial ALS index patients was compared with data from 827 control individuals, and significant enrichment of loss-of-function variants of NEK1 (0.57) was shown in FALS group compared with the control group (0.06), whereas mutations in known ALS genes were not detected in those patients carrying NEK1 mutations [42]. Based on this, NEK1 was suggested to be a novel familial ALS gene and other groups started to report similar results corroborating this idea. In a rare variant burden (RVB) analysis of a whole-exome from index FALS cases, a higher mutation profile of NEK1 was found in FALS and SALS cases [41]. Similarly, in a sample of Chinese patients, whole-exome analysis contributed to European evidence showing that NEK1 is an ALS gene as well as reporting new variants [43]. Furthermore, given the few studies endorsing NEK1 as an ALS gene and the ethnic heterogeneity of genes from ALS patients, Shu et al. [44] published a mutation screening of NEK1 from Chinese ALS patients by Polymerase Chain Reaction (PCR)-sanger sequencing, which led to the identification of coherent data of risk variants of NEK1 and, in total, three novel heterozygous loss-of-function mutations were identified.

Additionally, it is worth mentioning that Nguyen et al. [45] conducted a cohort study and characterized the genetic viability of NEK1 in ALS and frontotemporal dementia ALS (FTD-ALS) patients. Finally, it was demonstrated that the DNA damage accumulation was activated by variants of NEK1 in motoneurons derived from patients [46]. Other ALS-genes were also related to DDR mechanisms, such as FUS1 and C9orf72, which supports the idea that NEK1 plays a role in ALS, since it was also demonstrated to be involved in the DDR.

NEK1 has been suggested to play a role in the DDR, since the first protein interactome study revealed that NEK1 interacts with marker proteins of the DDR, including RAD54/ATRX and MRE11 among others [47]. Later, functional studies by other groups confirmed that NEK1 acts upstream of ATR/ATRIP [48], priming this complex for effective DNA damage signaling. Furthermore, a single serine residue in the RAD54 amino acid sequence (Ser572) is the target of NEK1, and its phosphorylation is key for the orchestration of homologous recombination DNA repair and the promotion of replication fork stability [49].

Ciliopathies are a group of multi-organ ciliary dysfunction phenotypes associated with defective proteins that lead to abnormal function of the cilia [50,51]. As mentioned before, NEK1 was first described to be involved in primary cilia maintenance in a knockout experiment in mice, where its involvement in PKD development was described. In fact, its involvement has already been shown in human ciliary dysfunction diseases. Mutations in NEK1 can cause Short-Rib Polydactyly Syndrome (Type Majewski) [8], short-rib thoracic dystrophies [52], oral-facial-digital syndrome type II (Mohr syndrome) [53], and, more recently, short-rib polydactyly syndrome (SRPS) [54]. NEK1 mutations in intron 4 have already been associated with SRPS prenatal diagnosis [55].

Cancer is a group of more than a hundred diseases that are characterized by the abnormal proliferation of cells that affects any tissue of the body. Due to its complexity, the causes and development of the disorder were organized in hallmarks by Hanahan and Weinberg in 2000 [56] and restructured in 2011 [57]. In this context, proteins involved in processes related to the hallmarks of cancer are more likely to be involved in occurrence, progression, and aggressiveness. Many proteins, like cyclin-dependent kinase (CDK), are known to be the main regulators of cell cycle progression and, consequently, are related to neoplastic cells [58]. Moreover, an increasing amount of evidence is suggesting novel proteins for cancer research, such as NEKs [4].

Gliomas are the most common type of brain tumor; it develops from glial cells. Additionally, gliomas are classified in grades from I to IV to determine the stage of the disease, among which stage IV, also called glioblastoma multiforme (GMB), has a very poor prognosis [59]. Zhu et al. [60] reported a pattern of NEK1 overexpression in high-grade glioma tissues and cell lines. Furthermore, knockdown of NEK1 by siRNA inhibited glioma cell growth and increased susceptibility to temozolomide (TMZ), the main chemotherapeutic drug used to treat glioma, leading to apoptosis. Together, these results suggest an important role of NEK1 in human gliomas and TMZ resistance.

Wilms Tumor is the most common type of renal cancer in children and infants. This disorder has a complex etiology and leads to huge genomic alterations including copy number variants (CNV), and identification of those regions may give a better comprehension of cancer development. Cardoso et al. [61] revealed, by Microarray Comparative Genomic Hybridization (aCGH), CNV is related to potential biomarkers for cancer development, including deleted regions from the q arm of chromosome 4, where NEK1 is located. The relation between NEK1 deficiency and kidney development has already been demonstrated, as shown briefly in this review, but whether it is related to cancer development requires further investigation, for example, by assessing the dynamics of the interaction between CTNNB-1 and NEK1.

The Von Hippel Lindau (VHL) protein is a well-known tumor suppressor protein which is associated with elongins B and C, cullin 2, and Rbx1, which form an E3 ligase [62] for the proteasomal degradation of hypoxia-inducible factors (HIFs) under normoxic conditions. Loss of function mutations in these proteins lead to VHL syndrome, which is characterized by the formation of multiple tumors and cysts throughout the body. Moreover, VHL is also related to other cellular functions, such as cell proliferation, extracellular matrix deposition, and regulation of the primary cilia, which NEK1 is also known to regulate. This congruent activity led to a study regarding the interaction between VHL and NEK1 [63].

The study demonstrated a clear connection between those two proteins by revealing the phosphorylation and regulation of VHL by NEK1 via proteasomal degradation. On the other hand, Chen et al. (2019) published a recent study demonstrating the regulation of NEK1 by VHL via HIF-2α and the ubiquitin-proteasome pathway in renal cancer cells. They demonstrated that NEK1 is up-regulated in ACHN and Caki-1 cells and down-regulated in the VHL-deficient 786-O, 769-P and A498 cells, at both the protein and mRNA levels. Additionally, the presence of a Hypoxia Induced Element (HRE) at the NEK1 promoter and specific binding to HIF2-α suggested that NEK1 may be a novel hypoxia-induced gene, which was confirmed under hypoxic conditions. Conversely, data from those two studies suggest that there may be positive feedback between VHL and NEK1 and a potential pathway for cancer and ciliopathy research [64].

Recently, NEK1 has been linked to prostate cancer (PCa) by its function in DDR. Singh et al. published two studies in a row demonstrating the role of the TLK1/NEK1 axis in PCa progression [65] and its pharmaceutical potential as a novel therapy combined with standard treatment [66]. The combined strategy of standard androgen-deprivation treatment (ADT) and thioridazine (TDH), a specific inhibitor of Tousled Like Kinases (TLK), inhibited an increase in the TLK1B prosurvival mechanism from PCa cells, leading to apoptosis. These results led to further investigation of this PCa adaptation to ADT. The TLK1/NEK1 axis is one of the earliest events in PCa adaptation and progression to androgen-independent growth.

In a recently published paper by our group, a direct link between NEK1 and thyroid cancer was observed [67]. Tissue microarray analysis revealed increased expression of NEK1 in classical and follicular variants of papillary thyroid cancer, allowing us to use these levels of expression to discriminate the malignancies with high specificity and sensitivity. High expression of NEK1 was also observed in tumors with multifocality and in patients with lymph node metastasis, associating NEK1 with a poor prognosis.

## 3. NEK2

Human NEK2 is the most closely related to NIMA (Never in mitosis A) and very is well described in the literature [7]. NEK2 is regulated by the cell cycle, and high levels of NEK2 were found in the S and G2 phases, and low levels were found in the M and G1 phases [9]. Furthermore, the NEK2 protein is essential for centrosome integrity and plays a role in the regulation of centrosome separation [20]. By employing immunofluorescence microscopy and biochemical fractionation, NEK2 was found in the centrosome. Centrosomes from KE37 human leukemic cell lines were isolated by fractionation and then analyzed by immunofluorescence microscopy and Western blotting. Immunofluorescence microscopy showed that NEK2 is associated with centrosomes regardless of their position in the cell cycle. In addition, NEK2 localizes to the centrosome throughout the cell cycle, and its kinase activity and overexpression promote the centrosome split into fragments diffuse [20]. Many studies in the literature display the relationship between disturbances in the centrosome and cancer progression [68].

Fry et al. (2002) [69] identified two alternative splice variants, NEK2A and NEK2B. Both genes are localized in chromosome 1. The overexpression of the NEK2A variant is able to induce centrosome splitting. Moreover, during mitosis, the expression of each variant depends on the cell cycle [69]. NEK2 was found in the nucleus during the S phase, and it was found in the cytoplasm at the midbody in the telophase, although changes in NEK2 expression throughout the cell cycle have been reported. Furthermore, the association with chromosomes occurs during the passage from prophase to metaphase, and dissociation occurs during anaphase [70]. NEK2 is involved in different diseases. A considerable number of studies linking NEK2 to cancer and other disorders have been published in the literature, and these are presented in this review.

Left–right (LR) asymmetry may cause birth defects in humans during embryogenesis, this process can be related to the cilia. For example, the misalignment of the left and right sides of the cardiovascular system can cause birth defects [71]. NEK2 is related to ciliary function. Endicott et al. employed morpholino technology to produce NEK2 knockdown in Xenopus embryos, the NEK2 knockdown caused abnormally looped hearts. The authors observed that the cilia and motile cilia counts were in *nek2* morphants and hNEK2 overexpressors. Additionally, NEK2 overexpression promotes increased cilia resorption by phosphorylation of Nup98 [24]. 

NEK2 has been shown to be related to malignant gliomas, which are highly invasive. This category consists of glioblastomas, astrocytomas, oligodendrogliomas and oligoastrocytomas [72]. These tumors are associated with poor prognosis and poor quality of life [73]. Liu et al. (2017) [74] selected 99 patients, and the analysis of these gliomas indicated that NEK2 was highly expressed compared with non-tumorous brain tissues. High levels of NEK2 were also related to poor patient overall survival [74]. Another group revealed the specific relationship between NEK2 and glioblastoma multiforme (GBM) [75], also known as glioblastoma or grade IV astrocytoma. GBM is the most common, aggressive, and frequent type of malignant brain tumor that affects humans and treatment is difficult to perform. Furthermore, patient survival is 14–15 months [76]. It is well known that the enhancer of zeste homolog 2 (EZH2) is overexpressed in GBM cells [77]. NEK2 forms a complex with EZH2 and regulates its post-translation modification, protecting EZH2 from degradation by ubiquitin-proteasome. Along with this, the authors correlated high expression of NEK2 with poor patient prognosis, and after therapy, the level of NEK2 was found to increase compared to primary untreated tumors. The authors concluded that NEK2-EZH2 could be a target for drug development [75].

Multiple myeloma (MM) is a type of cancer that affects the plasma cells. It is the second most prevalent type of hematological cancer. This cancer predominantly affects patients over 65 years old [78]. For MM, drug resistance remains a key problem in cancer treatment [79]. By employing the tandem affinity purification-mass spectrometry (TAP-MS) technique, coimmunoprecipitation, and pull-down assay, Franqui-Machin and coworkers found that, in MM cells the deubiquitinase USP7 interacts with NEK2 [80]. In that study, the authors verified that the complex NEK2-USP7 protects NEK2 from degradation by the ubiquitin proteasome, which could improve drug resistance in MM. Additionally, the interaction between USP7-NEK2, leading to the deubiquitination and stabilization of NEK2, was confirmed [80]. Moreover, high expression of NEK2 was observed in MM cells with an increase in focal bony lesions [81].

Colorectal cancer (CRC) is one of the most common cancers diagnosed in humans and is an important cause of mortality worldwide. The accumulation of mutations in epithelial and preneoplastic cells, which is thought to be linked to a combination of lifestyle habits and genetic factors such as smoking, aging, diet, and obesity, is probably a major cause of the development of this cancer [82].

Lu and colleagues (2015) [83] demonstrated high levels of NEK2 in colon cancer tissue by comparing paracancerous and normal tissues. They analyzed the level of NEK2 expression in 60 colon cancer samples, 30 paracancerous colon tissue samples and 10 normal colon tissue samples. The paracancerous tissues had high levels of NEK2 compared with normal colon tissues. Interestingly, poor prognosis of the patients was related to high levels of NEK2 [83].

Several published studies have focused on the overexpression of NEK2 in hepatocellular carcinoma (HCC). HCC is a primary human liver cancer that has been reported worldwide [84]. It is more prevalent in males than females. The risks for HCC development include chronic liver disease and cirrhosis [85].

One of these studies compared HCC tissue samples from patients with adjacent nonmalignant tissues, and it was observed that the level of NEK2 protein was higher in human HCC tissue. Moreover, the expression of NEK2 was analyzed in HepG2 and six other cell lines (HL-7702, PLC/PRF/5, Hep3B, BEL-7402, SMMC-7721, and QCY-7701). Compared with controls, NEK2 was again overexpressed in HCC cells. By employing NEK2 siRNA, the cell viability declined compared with the control groups. In addition, the depletion of NEK2 in HepG2 cells induced cell cycle arrest in the G2/M phase. Furthermore, the authors propose that NEK2 regulation contributes to HCC growth by the β-catenin/Wnt pathway [86]. Other groups studied the relationship of NEK2 expression in 100 HCC patient tissue samples that received liver transplants. Immunohistochemical assay data showed high NEK2 expression in 69% of the samples. Furthermore, HCC patients with high levels of NEK2 presented a poor prognosis. miR-486-5p was detected to be a NEK2 upstream negative regulator and thus was considered a potential target for drug therapy [87]. It is assumed that microRNAs can regulate gene expression and have an important role in cancer progression [88]. Additionally, through immunohistochemistry analyses, Lin and coworkers (2016) revealed high levels of NEK2 in HCC patients. Additionally, patients positive for NEK2 had a poor prognosis compared with negative controls. Furthermore, their results showed that NEK2 knockdown inhibited CSC-like properties in HCC, including self-renewal, chemotherapeutic resistance properties, and downregulation of the expression of stemness associated-genes of CSCs, such as Nanog, Sox2, Bmi-1, EpCAM, CD133, K19, LIN28, and NOTCH1 [89]. Corroborating those studies, another group found high levels of NEK2 expression in human hepatocellular carcinoma HCC tissues and cell lines, which was correlated with clinical progression indicators such as tumor size, differentiation grade, and lymph node metastasis. In addition, the authors demonstrated that high levels of NEK2 contribute to HCC progression and drug resistance by PP1/Akt and Wnt pathways [90]. Li et al. (2016) [91] associated the upregulation of NEK2 with poor prognosis of 63 patients with HCC from 2010 to 2013. Moreover, high levels of NEK2 were correlated with the tumor nodule number and recurrence, and with the expression of phospho-AKT (V-akt murine thymoma viral oncogene homolog) and MMP-2 (72 kDa type IV collagenase also known as matrix metalloproteinase-2) [91]. After hepatectomy, liver non-capsulation and poor survival outcomes in HCC patients were related to NEK2 overexpression. The epithelial-mesenchymal transition of HCC is induced by NEK2 and it consequently promotes HCC invasion. In addition, HCC cell lines like HepG2, BEL7402, Hep3B, SMMC7721, PLC/PRF/5, and HCCLM3 presents high levels of NEK2. Analyses of RTqPCR showed that, levels of NEK2 mRNA from HCC tissues increased when compared with those normal liver tissues [92]. Finally, NEK2 overexpression contributed to HCC tumor growth, migration, and angiogenesis by pAKT/NF-κB (factor nuclear kappa B). Regarding angiogenesis, NEK2 also induced IL-8 (Interleukin 8) expression [93].

Melanoma which is considered a very aggressive tumor that affects the melanocytes, is also related to NEK2. The lesions can be found throughout the body (including in the skin, iris, and rectum), and the major cause is ultraviolet radiation exposure [94]. Every year, this cancer affects tens of thousands of people worldwide, being the most dangerous type of skin cancer [95].

Like the other cancers described before, high levels of NEK2 were found in melanoma tissues, and the overexpression of NEK2 is related to poor overall survival and loss of p53 [96].

Pancreatic cancer is the 11th most common cancer. In 2018, there were 432,242 cases of pancreatic cancer worldwide [97]. Obesity, family history of chronic pancreatitis, diabetes mellitus, a non-O blood group, advanced age, smoking, and male sex are major risks factors for this type of cancer [98]. NEK2 overexpression is also related to pancreatic cancer. The depletion of NEK2 by siRNA was found to inhibit pancreatic cancer tumor growth in a xenograft mouse model. High levels of NEK2 were also found in eight pancreatic cell lines: KLM1, KP4, Panc1, PK45H, PK8, PK9, and MIA PaCa-2. By employing siRNA by a portal venous port–catheter system, the authors observed that the tumor volumes of NEK2 siRNA-treated mice were significantly lower, and the survival time was prolonged. The area and the number of liver metastases reduced after NEK2 siRNA treatment [99].

Nasopharyngeal carcinoma (NPC), also related to NEK2, affects the nasopharynx epithelium. Frequently, NPC affects the fossa of Rosenmuller [100]. Each year, 80,000 cases of nasopharyngeal carcinoma are diagnosed worldwide. There is a strong relationship between Epstein-Barr virus (EBV) and NPC [101]. Overexpression of NEK2 is related to advanced clinical stages (III and IV) of NPC. In addition, NEK2 overexpression is related to poor overall survival for patients with NPC and, promotes cancer cell proliferation by reducing cellular apoptosis. Additionally, NEK2 contributes to cisplatin resistance [102].

Around the world, about 1.8 million new cases of lung cancer are diagnosed each year [103]. Since the mid 1960s, it has been known that the major cause of lung cancer is tobacco smoking [104]. Shi et al. (2017) [105] demonstrated the relationship between NEK2 and lung cancer. The overexpression of NEK2 in lung cancer patients compared with that in normal subjects has been reported, and the upregulation of NEK2 is related to poor overall survival, relapse-free survival, and increased risk of recurrence [105]. 

NEK2 is related to breast cancer [106]. Breast cancer is the most common type of cancer in women. Along with lung and colon cancers, breast cancer is one of the most prevalent types of cancer worldwide. Thanks to early detection and efficient therapies, deaths caused by breast cancer have decreased in North America and the European Union [107]. However, it is estimated that there were 268,670 new cases of breast cancer in 2018 in the United States, with 41,400 deaths [108]. Hayward and colleagues demonstrated an increase in NEK2 expression in breast cancer tumors. Additionally, they showed an elevated expression of the NEK2 protein in breast, ovarian, leukemia, prostate, and cervical cancer cells. Regarding breast cancer, the authors analyzed 20 human breast cancers, and it was found that NEK2 overexpression was present in the majority of these samples. Overexpression of NEK2 was detected in ductal carcinoma in situ (DCIS) tumors, invasive ductal carcinoma, and invasive lobular carcinoma [106].

Centrosome amplification is associated with cancer and is considered the major factor contributing to the development of this disease [109]. Breast adenocarcinomas have high rates of frequency defects on the centrosome [110], and it is known that NEK2 is a centrosomal protein [111]. Marina and Saavedra (2014) showed that NEK2 overexpression in breast tumor samples is related to poor prognosis. Additionally, NEK2 is overexpressed in Her2-positive breast cancer cells with centrosome amplification [112].

Another study showed that centrosome amplification can be modulated by CDK4 and NEK2. CDK4-depleted Her2-positive cells (breast cancer cells) abolish centrosome amplification and binucleation. A decrease in NEK2 protein levels in CDK4 knockdown cells was observed. The authors suggested a relation between CDK4 and NEK2 [113].

Telomeric repeat binding factor 1 (TRF1) plays a role in telomere maintenance and the cell cycle [114,115]. NEK2 interacts and phosphorylates TRF1 in vivo and in vitro. The interaction between NEK2 and TRF1 was analyzed by the immunoprecipitation of MCF7 (breast cancer) cells, and the association of both proteins was higher in the G_2_/M phase. In addition, using immunofluorescence microscopy analyses, NEK2 and TRF1 were shown to be co-localized throughout the interphase and prophase. During the prophase, both proteins localized to the condensed chromosomes. Breast cancer cells, MDA-MB-231, and MCF7, were treated with TRF1 siRNA, and NEK2 was overexpressed concomitantly. The TRF1-depleted cells with NEK2 overexpression presented normal centrosome numbers. The authors also observed an increase in the numbers of multinucleated cells associated with overexpressed NEK2 cells, and these numbers decreased in TRF1-depleted cells. High levels of NEK2 were shown to cause abnormal spindle pole numbers and chromosomal misalignments, but, in the presence of siRNA TRF1, the abnormalities were attenuated [116].

Other groups also associated NEK2 with breast cancer. Capello et al. demonstrated the role of NEK2 in breast cancer. Patients with breast cancer were selected and the levels of NEK2 mRNA were accessed. High levels of NEK2 mRNA were found in those patients, which was correlated with a poor prognosis and breast cancer recurrence. Moreover, high levels of NEK2 protein expression were detected in breast cancer patients. After injecting MDA-MB-231-shNek2 cells or controls in mice, a decrease in the tumor burden was observed in animals that received MDA- MB- 231-shNek2 cells, and this could be related to migration and invasion deficiency. Furthermore, analyses in breast cancer cells showed that the depletion of NEK2 induces aneuploidy, alteration in the centrosome, deregulation in the cell cycle, and increased apoptosis [117].

Furthermore, the overexpression of NEK2 is associated with obese patients with luminal A breast cancer [118]. In summary, NEK2 has the potential to be used in drug development and cancer therapy. 

## 4. NEK3

Human NEK3, a 56 kDa protein, has 489 amino acid residues, and its gene is localized to 13q14.2 [119]. NEK3 has an N-terminal catalytic domain and a C-terminal regulatory domain but no coiled-coil regions [120]. Murine NEK3 expression was found in the cytoplasm, and the NEK3 levels were found to vary through the cell cycle. NEK3 expression is higher in quiescent cells during the G0-arrested cell cycle. However, NEK3 activity inhibition mutations, and overexpression did not affect cell cycle progression [10]. High levels of NEK3 were reported in neurons from the central and peripheral nervous systems in the cytoplasm and axons [121].

Data show the role of NEK3 in breast cancer and gastric cancer. 

Serine/threonine NIMA (Never in Mitosis A)-related family kinase NEK3 is involved in breast cancer development. A study showed the interaction between NEK3 and Vav1 and Vav2 using immunoprecipitation analyses. Using T47D cells, prolactin(PRL) stimulation leads to NEK3 activity and its interaction with Vav2 and PRL receptors. Furthermore, NEK3 contributed to Vav2 phosphorylation [122] and was shown to interact with the key regulator RhoGDI2, which is involved in the same signaling pathway [123]. 

The activation of the Vav family is related to prolactin (PRL) receptor activation, which contributes to the development of human breast cancer [124]. In addition, NEK3 has been described as having a role in PRL-mediated cytoskeletal re-organization and breast cancer cell migration and invasion. By employing breast tissue microarrays, NEK3 overexpression was observed in malignant specimens compared with normal tissue [125].

Worldwide, gastric cancer (GC) is the fifth most common cause of mortality [126]. Stomach cancer is related to lifestyle, genetic factors, alimentary habits, and *Helicobacter pylori* (*H. pylori*) infection [127]. Tobacco smoking and alcohol consumption also increase the risk of developing GC [128]. NEK3 is also related to human gastric cancer development. NEK3 overexpression in gastric cancer tissues has been reported when compared with normal tissues, and this is related to poor overall survival. High levels of NEK3 are associated with pathologic stage, lymph node metastasis, and poor prognosis of GC. Additionally, NEK3 overexpression is related to poor overall survival [129]. Similarly, an analysis of papillary thyroid carcinomas and follicular thyroid carcinomas revealed that the expression of NEK3 is stronger in tall-cell papillary thyroid cancer, with the highest levels being observed in patients with metastases [67].

## 5. NEK4

The clear function and involvement of NEK4 in cellular processes are yet to be determined, but it is suggested that NEK4 participates in primary cilium assembly, regulation of microtubule stabilization [25], the DNA damage response (DDR) [31], and RNA splicing [18]. A second functional isoform [18], which is similar to the murine NEK4 long isoform and named NEK4.2, has been identified in humans, and different interactors to NEK4.1 have been described. Given the potential involvement of NEK4 in ciliary function and the DNA damage response, the association with human disorders is to be expected. However, there is little data published in this context, and further studies must be conducted to confirm whether there is a clear connection between NEK4 and human diseases.

NEK4 interacts with RPGR-interacting protein 1 (RPGRIP1) and RPGRIP1-like protein (RPGRIP1L) and may function as a scaffold to recruit NEK4, which regulates cilium stability [130]. This interaction profile might be involved in ciliopathies, and NEK4 down-regulation may influence the susceptibility to those disorders. A genomic approach in patients with ciliopathy phenotypes [131] led to the identification of seven novel candidates genes linked to ciliopathies, including loss-of-function mutations in NEK4.

Given its possible cellular functions, NEK4 may also be involved in cancer progression. NEK4 appeared to be also connected to tumor necrosis factor-related apoptosis-inducing ligand (TRAIL) resistant cancer cells. TRAIL is a member of the TNF family that triggers apoptosis and preferentially targets cancer cells, which turns it into a target for cancer therapies. It was revealed that NEK4 is overexpressed in lung and colon cancer tissues, and its loss-of-function sensitizes TRAIL-resistant cells to cell death in vitro and in vivo, using mouse xenograft models [132].

In this context, two more studies relating NEK4 to cancer development were published. First, NEK4 was identified to be differentially expressed across the four stages of colorectal cancer (CRC) [132], showing that the higher the stage of CRC is the lower the level of NEK4 expression is. The analysis also demonstrated that NEK4 has the strongest *p* adjusted levels; therefore, it has the highest level of significance among the investigated genes. In the lung cancer context, NEK4 was also demonstrated to regulate the epithelial to mesenchymal transition (EMT), which is a cellular process that occurs in cells that lose their epithelial phenotype and acquire mesenchymal characteristics, and has been linked to cancer cell migration and invasion [133]. In the study, siRNA library screening in lung cancer cells expressing the E-cadherin promoter-reporter construct revealed that NEK4 is a positive regulator of lung cancer EMT, resulting in increased potential for cells to migrate and invade.

## 6. NEK5

Although NEK5 is the least characterized kinase of the group, as its functions are being elucidated, its role in human disorders is becoming clearer, demonstrating its potential for pharmacological approaches. The first physiological role described for NEK5 was its involvement in myogenic differentiation through the regulation of Caspase-3 [27]. NEK5 was also found to regulate centrosome integrity in the interphase [21]. Most recently, NEK5 was linked to DDR and genomic stability, where it was demonstrated that it interacts with topoisomerase II β (TOPOIIβ) [32].

Given its relationship with cell cycle progression, NEK5 may also be a potential cancer biomarker. In 2011, the correlation between the NEK family and cancer was demonstrated [4], but no recording of NEK5 was characterized. This did not occur until 2017, when, for the first time, NEK5 was shown to be overexpressed in an RNA-seq [134] analysis of Russian patients with benign prostatic hyperplasia (BPH) and prostate cancer (PCa), and this was further validated by RTqPCR. In addition, Pei et al. demonstrated the dynamics of NEK5 in breast cancer cell proliferation [135]. In that study, NEK5 was shown to be overexpressed in breast cancer tissues, suggesting that it can promote the growth of breast cancer cells, whereas the mechanism underlying its effects can be explained by its interaction with, and up-regulation of, cyclin A2. An analysis of thyroid tissues revealed a high expression of NEK5 in the tissues of patients with invasion and metastasis. Additionally, the expression of NEK5 was related to the patient’s tumor size, showing higher expression with an increase in the tumor size [67].

To date, there have still been few studies relating NEK5 directly to human diseases, yet the biological functions already described reveal its potential for use in pharmacological approaches. However, this requires further investigation. That being said, full understanding of its mechanisms of action and interaction profile may indicate the direct involvement of NEK5 in human disorders and may also reveal novel treatments.

## 7. NEK6

NEK6 is a serine/threonine kinase with 313 amino acids that is encoded in humans by the *Nek6* gene in chromosome 9. NEK6 comprises a short disordered N-terminal region, which is essential for its interaction with cellular partners, and a catalytic domain at the C-terminal [136]. Its globular and monomeric conformation, although slightly elongated, was revealed by Meirelles et al. (2011) through size-exclusion chromatography-multi-angle light scattering (SEC-MALS) and small-angle X-ray scattering (SAXS) experiments, together with molecular modeling [137]. The subcellular location of NEK6 is diffuse in the cytoplasm. It may be present in the nucleus, in the centrosome during metaphase and anaphase, and in the midbody in cytokinesis [11]. The expression and activity of NEK6 increase during mitosis, wherein the activity of NEK6 is dependent on its phosphorylation at serine_206_ by NEK9. Activation of NEK6 during mitosis is important for the formation and maintenance of the mitotic spindle [14].

The activation of NEK6 is not only caused by direct phosphorylation of NEK9 but also by interaction with the noncatalytic C-terminal domain of NEK9, with consequent dimerization of NEK6 structures and inhibition of self-inhibitory residues (tyrosine 108) [138]. NEK7 also has a similar mechanism of kinase activation, which is described in the appropriate section. Thus, self-inhibitory residues can provide a potential target for selective inhibitors of such kinases.

In recent years, altered expression and activity of NEK6 were associated with several types of cancer, such as liver [139], prostate [140], gastric [141,142,143], colorectal [144,145], breast [146], serous epithelial ovarian [147], thyroid [148], and retinoblastoma cancers [149]. In addition, inflammation-based diseases, such as ulcerative colitis, have also been linked to NEK6 [28]. 

Increasing evidence suggests the involvement of NEK6 in the progression of hepatocellular carcinoma (HCC). Zhang et al. (2014) showed that NEK6 is upregulated in 79.1% of HCC patients, and it presents significantly increased expression in the Huh7, HepG2, Hep3B, and PLC/PRF/5 cell lines. Furthermore, NEK6 overexpression was shown to increase the proliferation and viability of HCC cells, while its silencing had the opposite effect, in vitro. The same study revealed that NEK6 silencing induced G2/M phase arrest and delayed the G0/G1 cell cycle phase in Hep3B cells, since NEK6 is involved in mitotic cell cycle progression. Additionally, NEK6 promotes cell proliferation and hepatic tumorigenesis by modulating the Cyclin B protein levels; this process is mediated by increasedCDC2 expression [139].

A relevant study showed that the NEK6 signaling pathway is the main mechanism responsible for castration resistance in prostate cancer (CRPC). Using a lentiviral library, the participation of 601 kinases in the development of subcutaneous androgen-dependent tumors was tested. NEK6 was reported to be a central kinase that is responsible for the development of tumors in the absence of androgens. The overexpression of NEK6 has been described in castrated males and female animals, representing absent systems of androgen hormones, resulting in increased tumor formation. Conversely, it was also reported that the reduction of NEK6 expression reestablished castration sensitivity. The study suggested that NEK6 may be related to cell survival pathways since no significant changes in proliferation or cell cycle progression were observed in NEK6 overexpressing prostate cancer cells [140]. 

Modifications of histones are well-characterized events in the development of cancer, especially in gastric carcinoma (GC). In a recent study, NEK6 and two other genes, *AURKA* and *HDAC2*, were found to be significantly overexpressed in patients with gastric carcinoma [141]. Another study associated NEK6 up-regulation in gastric cancer with distant and lymph node metastasis. Conversely, NEK6 down-regulation was able to reduce the migration and proliferation of cancer gastric cells [142]. Takeno et al. (2008) identified a significant increase in NEK6 expression in the samples of gastric cancer patients compared with controls, revealing that NEK6 may be a potential target for gastric cancer treatment [143].

The main risk factor for the development of colorectal adenocarcinoma (CRC) is the emergence of colorectal adenomatous polyps (CRAP), which are benign tumors that can progress to cancer through various genetic and epigenetic changes [144]. Kasap et al. (2016) identified that *NEK6* and other genes, such as *AURKA*, *AURKB*, *HDAC1,* and *PAK1*, are significantly overexpressed in samples from patients with CRC and CRAP. In addition, larger diameter polyps showed greater overexpression of NEK6 compared to smaller polyps [145]. All these findings show that NEK6 may be a predictive marker that can be used to determine the time of surveillance between the removal of polyps and the next colonoscopy. More studies are needed to better understand the role of NEK6 in CRC carcinogenesis.

Another important risk factor for CRC development is ulcerative colitis (UC). Gerçeker et al. (2015) identified NEK6 overexpression in samples from patients with ulcerative colitis and CRC. NEK6 was found to be overexpressed in patients with more extensive colon involvement and long-term ulcerative colitis. It was suggested that NEK6 is involved in the early stages of inflammation associated with CRC development in UC patients [28]. Thus, NEK6 could be an early marker of CRC in patients with ulcerative colitis. More studies are needed to identify the inflammatory signaling mechanisms that are up and downstream to NEK6 in the development of CRC.

Recently, the involvement of NEK6 in breast cancer was identified by He et al. (2018). Non-tumoral breast tissues showed low or absent expression of NEK6 whereas in breast cancer tissues NEK6 expression was highly detected in the nucleus and, to a lesser extent, in the cytoplasm. The survival rates of patients with breast cancer were also evaluated in relation to NEK6 expression, and 69% of patients with high expression of NEK6 were shown to be alive versus 87% of those with low expression. In this study, the reduction of NEK6 expression was able to attenuate the proliferation and spheroid formation of breast cancer cells [146]. Thus, NEK6 may participate in the uncontrolled proliferation and growth of breast cancer cells. The mechanisms behind the roles of NEK6 in breast cancer still need to be elucidated.

Ovarian cancer is the deadliest cancer that affects women. This is especially deadly due to the lack of detection in the early stages, late onset of symptoms, and high resistance to chemotherapeutic drugs during tumor progression [150]. Serous epithelial ovarian cancer (SEOC) is the leading type of ovarian cancer. A combination of platinum and/or taxane drugs is used against this type of cancer [151]. However, a large number of patients develop resistance to these treatments, which is, in part, stimulated by the low oxygen concentration in the cell microenvironment. In this context, survival pathways are activated to create a cellular adaptation against the hostile environment, generating resistance to conventional treatment. HIF1A is a transcription factor that is well known for its role in resistance to chemotherapeutic treatments since its activation by hypoxia leads to the transcription of target genes that play roles in apoptosis inhibition and drug efflux activation [152].

Evidence suggests that NEK6 is a downstream target of HIF-1α in ovarian cancer. Donato et al. (2015) showed that HIF-1α knockdown reduces the expression of NEK6, and hypoxic conditions were shown to increase HIF-1α and NEK6 in ovarian cancer cells. A bioinformatic analysis showed the presence of HRE (Hypoxia Responsive Elements) motifs in 5′ and 3′ UTR of the *NEK6* gene, which may be important for NEK6 regulation by HIF-1α. Additionally, in two groups of patients, concomitant expression of HIF1A and NEK6 was detected [147]. These data show that NEK6 can be regulated by HIF-1α in hypoxic conditions in serous epithelial ovarian cancer and may also be related to resistance to chemotherapeutic treatment. 

Circular RNAs (CircRNAs) were recently discovered, and their biological importance has been increasingly demonstrated. Currently, it is known that circular RNAs can regulate gene expression and interact with proteins to determine cell behavior. Circular RNAs are formed by a process called back-splicing and do not have 5 ‘and 3’ ends, since the ends are covalently bound, forming a circular structure [153]. 

Chen et al. (2018) showed that the circular RNA of NEK6 is up-regulated in thyroid cancer, promoting the growth and invasion of cells. The circular RNA of NEK6 may increase the expression of Frizzled class receptor 8 (*FZD8)*, a constituent of the Wnt signaling pathway, since it binds and inhibits mRNA-370-3p, a tumor suppressor. Full understanding of circular RNA functions will be critical to verify their role in cancer, and more studies aimed at development of new treatments for cancer are needed [148].

NEK6 may also exhibit expression modulation through interaction with microRNAs. A recent study showed that MiR-506-3p directly targets NEK6 mRNA, decreasing its expression and leading to reduced cell proliferation, G0/G1 cell cycle arrest, and apoptosis in retinoblastoma cells. Thus, MiR-506-3p acts as a tumor suppressor through the regulation of NEK6 expression in retinoblastoma cells [149]. 

A recent study showed that NEK6 is regulated by long non-coding RNA homeobox A11 antisense RNA (HOXA11-AS) through MiR-506-3p in retinoblastoma [154]. HOXA11-AS can decrease MiR-506-3p expression. Thus, overexpression of HOXA11-AS reduces the expression of MiR-506-3p, which, in turn, fails to downregulate NEK6. In addition, NEK6 and HOXA11-AS are overexpressed in retinoblastoma tissues, while the tumor suppressor Mir-506-3p is downregulated; these findings agree with previous findings. 

## 8. NEK7

NEK7 is a serine/threonine kinase composed of 302 amino acids encoded in humans by the *Nek7* gene in chromosome 1. Souza et al. (2014) revealed that NEK7 is involved in different cellular processes, such as cell division, intracellular protein transport, and DNA repair [12]. Another study by Souza et al. (2015) showed that NEK7 is able to bind to and phosphorylate Regulator of G-protein Signaling 2 (RGS2), which seems to be important for the organization and function of the mitotic spindle [15]. 

NEK6 and NEK7 are the shortest members of the NEK family and have very similar structures. NEK7 is also phosphorylated at serine_195_ by NEK9, leading to its activation. NEK6, NEK7, and NEK9 are present in the same cell signaling cascade [14]. 

Another form of activation consists of NEK9 self-associated binding to NEK7, which causes back-to-back dimerization and a conformation change in its self-inhibitory residue (tyrosine 97) [155]. This event increases the basal kinase activity of NEK7, promoting its autophosphorylation and activation. 

Structural comparison showed that the C-terminals of NEK6 and NEK7 proteins share similarity of 86%, while the disordered N-terminal corresponds to only 26% [14,156]. NEK7 shares some redundant functions with NEK6, such as participation in mitosis, the establishment of the mitotic spindle, and cytokinesis [11]. Despite the high levels of similarity between these kinases, Souza et al. (2014) suggested that NEK7 does not have the same interactors as NEK6 and, therefore, participates in different cellular mechanisms [12]. 

Extensive knowledge exists about the organization of microtubules in cycling cells. However, little is known about the molecular mechanisms that permeate the basic configuration of microtubules in neuronal compartments, which are postmitotic cells. 

A proteomic study found that NEK7 was upregulated in differentiating neurons [157]. Curiously, downregulation of NEK7 expression was shown to lead to defects during neuron differentiation. Thus, it was identified that NEK7 activity is essential for the growth and branching of dendrites as well as for spine formation and morphology. NEK7 is able to phosphorylate the kinesin Eg5/KIF11, which promotes its accumulation in the distal dendrites. Together, these results show that Eg5 limits retrograde polymerization of microtubules, causing dendrites growth and branching. Therefore, it is important to note that NEK7 is a regulatory protein of the microtubule cytoskeleton in both cycling cells and postmitotic cells. Interestingly, NEK6 does not share that role with NEK7.

Mutations in microtubule cytoskeleton regulators are associated with neurodegenerative diseases and neurodevelopmental disorders. For example, several mutations in the *MAPT* gene, which encodes the microtubule-associated protein tau (TAU), are associated with Alzheimer’s disease, while mutations in the *SPAST* gene, which is responsible for coding the microtubule-severing ATPase spastin protein (Spastin) are also involved in neuronal dysfunction [158,159]. Since NEK7 has been shown to regulate the formation of neurons in the hippocampus, a region associated with memory, we suggest that NEK7 may be involved in disorders of the nervous system. Thus, studies need to be carried out to understand the role of NEK7 in neurodegenerative and neurodevelopmental diseases. Similar to NEK6, NEK7 is also involved in several types of cancer, such as hepatic cancer [160], squamous cell carcinoma of the head and neck [161] and breast cancer [162].

Zhou et al. (2016) found that NEK7 was significantly overexpressed in hepatocellular carcinoma samples compared with normal liver tissues. Additionally, hepatocellular carcinoma cell lines, such as HepG2, Hep3B, Huh7 and SMMC7721, presented high levels of NEK7 mRNA compared to LO2, a normal hepatic cell line. In addition, the high expression of NEK7 is correlated with the expression of Ki-67, which is a known marker of cell proliferation. The 5-year survival rate was significantly reduced in patients with high expression of NEK7 and Ki-67. The knockdown of NEK7 promoted the reduction of viability in HCC cell lines and diminished the tumor volume in xenograft mice [160]. These findings reveal that NEK7 may be a therapeutic target of HCC. 

A study has shown that the Whsc1 gene has great relevance in the development of squamous cell carcinoma of the head and neck (SCCHN) [161]. WHSC1 knockdown reduced cell growth and stimulated apoptosis in SCCHN cell lines. NEK7 is a direct downstream target of the *Whsc1* gene and is also regulated at the transcriptional level by WHSC1 through the dimethylation of histone H3. Both WHSC1 and NEK7 knockdown reduced the percentages of cells in the G2-M phase compared to the control. These results suggest that NEK7 activation by WHSC1 is an important regulatory mechanism for cell-cycle progression in SCCHN.

Evidence shows that the silencing of UNC45A, a member of a large family of myosin interactors, reduces cell proliferation in breast cancer in vitro and in vivo. Using a microarray analysis on Hs578T cells, UNC45A silencing selectively reduced NEK7 expression while other members of the NEK family did not present altered expression. NEK7 overexpression in HeLa and MDA-MB-231 cells, which have been silenced for the *UNC45A* gene, restored cell proliferation, showing that NEK7 is a key downstream protein of UNC45A that mediates cell proliferation in cancer cells. The study also showed that UNC45A regulates the function and structure of centrosomes through NEK7 [162].

NEK7 is also linked to NLRP3 inflammasome activation. The inflammasome is formed by a high molecular weight complex of proteins. Nucleotide oligomerization domain (NOD)-like receptor family pyrin domain containing 3 (NLRP3) is the main receptor, and it is capable of forming the inflammasome in response to various inflammatory stimuli. Upon activation, NLRP3 is able to interact with apoptosis-associated speck-like protein containing CARD (ASC), which recruits and binds to caspase-1, thus forming the NLRP3 inflammasome. Caspase-1 is cleaved upon activation and activates pro-inflammatory cytokines such as IL-1 and IL-18 and generates pyroptosis, a kind of inflammatory cell death [29].

Usually, NLRP3 inflammasome activation occurs through two signals. The first is known as priming, which is able to enhance NLRP3 expression mediated by NF-κB pathways that involve Toll-like receptors (TLR), nucleotide-binding oligomerization domain 2 (NOD2), and tumor necrosis factor receptor (TNFR). The second NLRP3 activation signal is related to the recognition of molecular sequences of pathogens known as pathogen-associated molecular patterns (PAMPs), such as bacterial RNA/DNA, bacterial toxins, and small antiviral compounds [163,164,165]. NLRP3 can also be activated by danger-associated molecular patterns (DAMPs), including reactive oxygen species, saturated fatty acids, ATP, uric acid crystals, and others [166,167,168,169]. The cytosolic efflux of potassium ions is also able to activate the inflammasome NLRP3 [170]. Schmid-Burgk et al. (2016) revealed that NEK7 is an essential component of NLRP3 inflammasome activation. Using genome-wide CRISPR (Clustered Regularly Interspaced Short Palindromic Repeats) screening, NEK7 was identified as an upstream component of the NLRP3 inflammasome [171]. A study published almost at the same time provided more details on the participation of NEK7 in the NLRP3 signaling pathway. Shi et al. (2016) demonstrated that in the presence of ROS, NEK7 binds to NLRP3, which is crucial for NLRP3/ASC interaction and for the next steps in inflammasome formation, such as the activation of caspase 1. NEK7 knockdown in mouse macrophages caused a profound reduction in IL-1β secretion in response to several stimuli related to NLRP3 activation like LPS, nigericin, and ATP. Despite the very similar structure of NEK6, this protein failed to interact with NLRP3, revealing a specific mechanism of NEK7 regulation. It was suggested by the authors that the involvement of NEK7 in mitosis and inflammation activation does not occur simultaneously [172]. Therefore, targeting NEK7 and NLRP3 interaction may be specific to the reduction ofpro-inflammatory agents that are elevated in NLRP3-mediated auto-inflammatory diseases.

Several other findings linking NEK7 with inflammation and inflammation-based diseases, such as diabetes, endometritis, systemic lupus erythematosus and various cancers, have been published [173,174,175]. Inflammation contributes to the development of type 2 diabetes, which triggers several serious complications, such as diabetic periodontitis in the oral cavity. Metformin, a drug used for diabetes treatment, was shown to reduce periodontitis symptoms as well as levels of IL-1β. These effects were accompanied by remarkable decreases in the expression levels of NEK7, NLRP3, caspase-1, and mammalian target of rapamycin (mTOR). The study also proved that NEK7 inhibition by metformin occurs independently of mTOR, since mTOR inhibition in RAW 264.7 cells did not alter NEK7 expression. These findings suggest that metformin is able to reduce inflammation in periodontitis through the inhibition of NEK7, and as a consequence, reduces the activation of the NLRP3 inflammasome, which ameliorates diabetic symptoms [175].

Retinopathy is another severe complication of diabetes. Mcc950, a potent inhibitor of the inflammasome, was tested for the first time in human retinal endothelial cells (HRECs) in a study by Zhang et al. (2017). As a result, Mcc950 was shown to reduce the activation of the NLRP3 inflammasome as well as 1L-1β secretion in HRECs. In addition, the treatment of Mcc950 did not affect NEK7 expression, but it was able to block the interaction between NEK7 and NLRP3, inhibiting inflammasome activity [173]. Mcc950 could potentially be used to treat inflammatory-based diseases and their complications, such as diabetic retinopathy.

Another study investigated the relationship between the NEK7-NLRP3 inflammasome and systemic lupus erythematosus (SLE). SLE patients exhibited down-regulation of NEK7, NLRP3, and ASC expression and up-regulation of caspase-1, IL-1β, and IL-18, when compared with healthy controls. After treatment with methylprednisolone for 2 weeks, peripheral blood mononuclear cells were collected for analysis. The levels of NEK7 and NLRP3 increased in treated SLE patients, while the expression levels of caspase-1, IL-1b, and IL-18 decreased [174]. In conclusion, the NEK7-NLRP3 inflammasome pathway may act in a protective manner in the pathogenesis of systemic lupus erythematosus. Since NLRP3 inflammasome activation depends on several factors and different molecules, further investigation is still needed to relate the evolvement of the inflammasome specifically to each disease.

## 9. NEK8

NEK8 is a serine/threonine kinase composed of 692 amino acids encoded in humans by the *Nek8* gene in chromosome 17. The literature is poor regarding NEK8 functions. NEK8 is present in the spindle poles, centrosome, and cytoplasm and may participate in mitotic spindle formation and centrosome separation [7]. NEK8 is also important for primary cilium stabilization; thus, ciliopathies are directly associated with NEK8. The kinase domain and the C-terminal non-catalytic domain homologous to RCC1 (regulator of chromosome condensation) of NEK8 are responsible for its localization in the cilia and centrosomes [7]. Established mouse models of polycystic kidneys have been shown to have mutations in the Nek8 gene. In these animals, the microtubule dynamics, mitotic spindle checkpoint and cytoskeleton are completely disturbed [176]. In summary, the literature shows that NEK8 is associated with diseases such as ciliopathies, including nephronophthisis (NPHP), polycystic kidney disease, and cancer [7,177,178,179]. Nephronophthisis (NPHP) is a renal ciliopathy characterized by end-stage kidney disease. More than 20 genes have been identified as triggers for this disease [180]. Two of these genes, *CEP164* and *ZNF423*, are related to the DNA damage response (DDR) [181]. Mutations in FAN1, a DDR protein, also appears to be related to degenerative renal disease [182]. The most characterized DDR protein, MRE11, was found to exist in a mutated form in another ciliopathy [183]. The DDR signaling pathway is essential for cells to detect DNA damage and trigger cell cycle arrest in order to correct the identified DNA damage, which is important for maintaining chromosome integrity. One important study contributed to the understanding of how NEK8, as well as its functions in DDR is linked to ciliopathies [33]. According to this study, NEK8 is essential for maintaining genome integrity. A lack of NEK8 induces H2AX phosphorylation in NEK8 knockdown HeLa cells and in NEK8 -/- mouse embryo fibroblasts (MEFs). Additionally, the comet assay displayed an increase in tail moment, showing evidence of double-strand breaks (DSBs) in these cells. After aphidicolin treatment, NEK8 knockdown cells presented deficient S phase progression, indicating that NEK8 is related to DNA replication. NEK8 also is important for replication forks. After investigating DNA fibers, the authors observed that IdU tracks are shorter in NEK8-knockdown cells. The decrease in the median interorigin distance in Nek8-/-MEFs implies the function of NEK8 in origin firing. Additionally, these cells present fork asymmetry. 

Along with those results, cyclin A-associated CDK activity is suppressed by NEK8, which is necessary to downregulate this activity following DNA damage. In addition, NEK8 interacts with ATR, and in the presence of aphidicolin, this interaction was shown to be stronger than without treatment. This implies that NEK8 participates in cell cycle checkpoints and may regulate the DNA damage response.

A lack of NEK8 is related to renal fibrosis and cyst formation. NEK8-depleted IMCD3 cells (spheroid) were submitted to immunostaining assay, and cilia were analyzed. The depletion of NEK8 affects ciliation formation. After aphidicolin treatment, the occurrence of cilia was reduced. In summary, replication stress, activation of CDK and depletion of NEK8 may contribute to the development of nephronophthisis (NPHP).

Another study highlighted the role of NEK8 in genomic stability through replication fork protection and homologous recombination (HR) [184]. NEK8 has been shown to perform these protective functions by regulating RAD51, an essential factor for homologous recombination and replication fork protection. 

Clinical manifestations, such as hypertrophic cardiomyopathy and renal dysplasia have been reported in individuals with heterozygous mutations in NEK8 [177]. Grampa et al. (2016) provided evidence that mutations in the *Nek8/Nphp9* genes trigger severe syndromic renal cystic dysplasia. These mutations have also been associated with extra-renal conditions, such as situs inversus, cardiomegaly, bile ducts, pancreas and brain defects, and a narrow thorax and short bowed femurs. *Nek8* missense mutations were shown to lead to defects in ciliogenesis, since reductions in the percentage of ciliated cells and the cilia length were detected. This may be explained as the mutation disabling the interaction between NEK8 and ANKS6 in a very important compartment of the cilia (INVS), resulting in defects in the integrity of the INVS. Mutations in NEK8 are also able to deregulate the Hippo pathway, which is crucial for the control of growth and organ size [177]. 

Ding et al. (2018) showed that NEK8 promotes gastric cancer cell proliferation using colony formation and migration in vitro assays. In vivo, NEK8 depletion inhibited gastric cancer cell growth. Additionally, high expression levels of NEK8 in gastric cancer patients are associated with poor survival rates. A direct interaction with the von-Hippel-Lindau tumor suppressor protein (pVHL), a known tumor suppressor, has been reported. The interaction leads to degradation and ubiquitination of the NEK8 protein, indicating a further tumor suppressor function of pVHL. This demonstrated that NEK8 has an important role in gastric cancer progression and its regulation by pVHL, and this finding has contributed to the development of new therapeutic strategies [178].

Another study showed an association between NEK8 and VHL in cancer cells [179]. Renal cancer cells with VHL defects presented a high expression of NEK8, suggesting that VHL may downregulate NEK8 in these cells. These data suggest that NEK8 is downregulated by VHL through the HIF pathway in order to preserve the primary cilia structure in human renal cancer cells.

DNA repair defects are directly related to the development of cancer and the response to chemotherapy treatments. As already mentioned, NEK8 plays an important role in the maintenance of genomic stability. Further studies should be conducted to better understand the involvement of NEK8 in cancer.

## 10. NEK9

NEK9, or NERCC1, was first described by Roig J. et al. (2002) [185], and it was identified by immunoprecipitation with NEK6 in HEK293 cells. NEK9 is a 120 kDa kinase made up of 979 amino acids. NEK9 is activated during mitosis, interacts with Ran GTPase, and is phosphorylated by CDC2 [7,185]. NEK9 is autoactivated in vitro depending on the phosphorylation of an activation loop at T210. In addition, NEK9 is activated in vivo during mitosis. In the early stages of mitosis, activated NEK9 was found to be located at the centrosomes and spindle poles, implying that NEK9 plays an important role in cell division and suggesting that NEK9 participates in the microtubule organizing function of centrosomes during mitosis [16]. NEK9 is responsible for NEK6 and NEK7 phosphorylation at their activation loop sites, activating both of these kinases in vitro and in vivo [14]. NEK9 also has an important role in centrosome maturation [186]. The key mechanism for the formation of two dense microtubules asters (MT) in cells that enter mitosis is the accumulation of γ-tubulin in the centrosomes. Sdelci et al. (2012) showed that NEK9 interacts and phosphorylates NEDD1 on serine 377, which causes the recruitment of γ-tubulin to the centrosome in mitotic cells [186] NEK9 needs to be phosphorylated by Plk1 to carry out the process mentioned above. Although NEK6 and NEK7 are downstream effectors of NEK9, γ-tubulin recruitment occurs independently of these proteins.

The role of NEK9 in regulating microtubules may be directly related to its involvement in some types of cancer. A recent study showed that EML1-ALK variant 3, an oncogenic fusion present in non-small cell lung cancers, allows the recruitment of NEK9 and NEK7 proteins to microtubules via the N-terminal EML4 microtubule-binding region, leading to microtubule stabilization and increasing cell migration [187]. Beyond that, an elevation in NEK9 expression was associated with a reduction in progression-free survival in EML4-ALK patients. NEK9 is related to some diseases, as described below. 

Meningioma is a tumor of the central nervous system (CNS) and is the most common primary brain tumor. For diagnosis and removal of the tumor, surgery is necessary, but radiation therapy can also be used [188]. NEK9 was associated with meningioma in a screening of all meningioma grades and normal meninges samples by LC-MS/MS. A total of 3888 proteins and 3074 phosphoproteins were identified and activated. NEK9 was shown to be up-regulated in grade I, II and III meningioma. These data were also validated by Western blotting analyses [189].

Skeletal dysplasias are genetic disorders that affect bone growth and cartilage. They are a heterogeneous group of more than 450 disorders [190]. NEK9 was also shown to be associated with skeletal dysplasias [191].

A study using single nucleotide polymorphism homozygosity mapping and whole-exome sequencing revealed the relationship between the homozygous stop-gain mutation in NEK9 (c.1489C>T; p. Arg497*) with skeletal dysplasia in two Irish traveler families. The patients presented fetal akinesia, shortening of all long bones, multiple contractures, rib anomalies, thoracic dysplasia, pulmonary hypoplasia, and protruding abdomens. A loss of full-length NEK9 (107 kDa) in the patient’s fibroblasts was reported. Furthermore, the patients’ fibroblasts presented decreased cell proliferation, a delay in cell cycle progression, and a reduction in the cilia number compared to control fibroblasts [191].

Another disorder related to NEK9 is nevus comedonicus (NC). NC is a severe and rare skin disorder with few cases described in the literature. Commonly, it affects the face, neck or chest regions. NC is characterized by slightly elevated papules with keratinous plugs in the center. This disease can be treated with 0.1% tretinoin gel with a topical glucocorticosteroid ointment (mometasone furoate) [192].

Levinsohn et al. (2016) reported the relationship between NEK9 and Nevus Comedonicus [193]. Whole-exome sequencing was performed on DNA isolated from the tissues and blood of individuals NC101, NC102, and NC103 [193].

## 11. NEK10

NEK10 is one of the least studied NEKs. The kinase domain is localized in the center of this protein, which is different from the other NEKs. In addition, a coiled-coil region is located near the kinase domain, and there are four armadillo motifs at the amino-terminal regulatory domain, which probably function in protein–protein interactions [7,38,194]. 

NEK10 participates in the cell cycle; more specifically, in the maintenance of the G2/M checkpoint followed by ultraviolet (UV) irradiation. NEK10 forms a complex with RAF1 and MEK1 and acts as a positive regulator of ERK1/2 (Extracellular signal-regulated protein kinases 1 and 2) after UV irradiation [13]. This ERK1/2 activation has been related to the checkpoint between the G2/M phases of the cell cycle [195,196] and apoptosis [195]. 

In addition to NEK2, a study in the literature showed that NEK10 is also related to melanoma [197]. The authors found a NEK10 E379K mutation in two patients from seventy-seven samples of Japanese primary and metastatic melanomas, including cases of unknown primary origin regarding their clinicopathologic manifestations [197].

Mutations in NEK10 are also associated with breast cancer development [198]. Briefly, 286 luminal cancers from The Cancer Genome Atlas (TCGA) consortium and a group of 84 Estrogen Receptor positive primary tumors of metastatic breast cancer patients treated with aromatase inhibitors were selected. NEK10 mRNA expression levels were analyzed and the relationship with the PIK3CA genotype was examined. NEK10 mRNAs were found to be related to PIK3CA mutations in both groups. In breast cancer, PIK3CA is the most frequent mutated gene [198]. Another study involving NEK10 and breast cancer was performed by Milne et al. (2014) [199]. In this study, 41 non-synonymous single-nucleotide polymorphisms (nsSNPs) associated with breast cancer were studied. A strong association was verified for NEK10 (NEK10-L513S at 3p24 (rs10510592, OR 5 1.10, 95% CI 5 1.07–1.12, P 5 5.1 3 10217)) [199]. Taken together, the studies described here suggest that mutations in NEK10 could be a biomarker for melanoma and breast cancer detection. 

Moreover, NEK10 has an important role in ciliogenesis in mammals [26]. NEK10 forms a complex with PKA and PCM1 and participates in the cAMP pathway in cilium formation. When NEK10 is phosphorylated by PKA at T812, CHIP leads NEK10 to ubiquitination and proteolysis, consequently leading to cilium disassembly [26]. 

## 12. NEK11

NEK11, as with NEK10 and NEK5, has not been well studied. Noguchi and coworkers (2002) [34] were the first to study NEK11. The C-terminal region has PEST-like motifs and coiled-coil regions, such as NIMA from *Aspergillus nidulans*. The NEK11 catalytic domain in the N-terminal has high similarity with the N-terminal from NEK3 and NEK4. In addition, NEK11 has two different isoforms termed 74 KDa-NEK11L (long) with 645 residues and 54 KDa-NEK11S (short) with 470 residues. Furthermore, NEK11 is a cell cycle-regulated protein, and increased NEK11 expression can be observed through the S to G2/M phases [34]. During the G1/S arrested phase, NEK11 and NEK2A are colocalized at the nucleoli, and biochemical approaches suggest that NEK2A can regulate NEK11 [200]. NEK11 is also linked with DNA replication and the DNA damage responses [34]. The depletion of NEK11 leads to defects in asymmetric cell division in meiotic mouse oocytes, with prevalent participation of the NEK11S isoform [19]. 

NEK11 is associated with colorectal cancer. Sabir and coworkers [201] demonstrated that NEK11 promotes cell arrest in G2/M in HCT116 WT (human colon carcinoma) after drug treatment. The depletion of NEK11 cannot prevent the cell arrest at the G2/M checkpoint, leading the cell to a mitotic catastrophe. NEK11 is downstream of ATM/ATR and Chk1, preventing cell cycle progression to mitosis [201].

Liu et al. (2014) [202] demonstrated the relationship between NEK11 and ovarian cancer. Using bioinformatics, the authors analyzed the mRNA expression, protein gene interaction, protein small molecule interactions, annotation of biological processes, and microRNA mRNA interactions. They concluded that, in 586 cases of ovarian serous cystadenocarcinomas and cisplatin-resistant A2780 ovarian cancer cells, NEK11 mRNA is downregulated. Furthermore, NEK11 was found to interact with 22 proteins and 4 small molecules related to drug resistance in ovarian cancer [202].

## 13. Pharmacological Inhibitors of the NEK Family

Considering that NEKs are targets for various diseases, several inhibitors have been described as having effects against NEKs. Table 1 presents and summarizes a list of compounds presenting effects against NEK-related diseases. The effects are presented regarding the selectivity of the inhibitors, showing the direct and indirect effects on NEKs. Given the participation of the NEK family in several diseases, it is of paramount importance to identify those possible therapeutic targets, aiming at new opportunities for prevention, diagnosis, and treatment.

## 14. Conclusions

In summary, NEKs are crucial proteins for vital processes in cells, such as the cell cycle, mitosis, cilia formation, and the DNA damage response. They participate in the differentiation of cells and maintenance of cellular homeostasis. There is a considerable amount of information in the literature regarding NEKs linking these kinases to several human diseases including neurodegenerative diseases such as ALS and schizophrenia, inflammatory diseases, dystrophies, and ciliopathies. Their involvement in cellular mechanisms related to the formation and progression of cancers, including lung, breast, prostate, ovarian, colorectal, pancreatic, hepatic, and gastric neoplasias, is also remarkable and may be considered for future therapeutic approaches. Several inhibitors have been described in pre-clinical research to have direct and indirect molecular effects on NEKs. However, more studies are required to specifically target NEK-related diseases by pharmacological approaches. This review sheds light on this neglected kinase family and highlights its members’ potential as therapeutic targets. 

## Figures and Tables

**Table 1:** Drugs and inhibitors of NEK family members.

Drug Name	Chemical Formula	NEK Targets	Action/Effects	Stage of Development	Selectivity	References
Fostamatinib	C_23_H_26_FN_6_O_9_P	NEK1 NEK3 NEK4 NEK5 NEK9 NEK11	Inhibits signal transduction by Fcγ receptors involved in the antibody-mediated destruction of platelets. NEKs 1,2,3,4 5,9,11 are included in the list of targets	Approved for the treatment of chronic immune thrombocytopenia (ITP). Under investigation for other diseases	The active metabolite of fostamatinib, named R406, presents a lack of selectivity among several kinases	[203]
5-[(*Z*)-(5-Chloro-2-oxo-1,2-dihydro-3H-indol-3-ylidene) methyl]-*N*,2,4-trimethyl-1*H*-pyrrole-3-carboxamide	C_17_H_16_ClN_3_O_2_	NEK2	Interacts with NEK2	Experimental	No off-targets have been described	[204]
NCI code 51,525 and 58991	-	NEK2	Inhibits NEK2	Experimental	No off-targets have been described	[205]
MBM-17 and MBM-55	C_28_H_29_N_6_ and C_28_H_28_N_6_O_2_F respectively	NEK2	Inhibits NEK2 and has antiproliferative/antitumor properties	Experimental	It has low nanomolar activity and a great selectivity for NEK2	[206]
di-demethylchlorpromazine and 2-[5-fluoro-1*H*-indol-3-yl] propan-1-amine	C_15_-H_15_-Cl-N_2_-S.Cl-H and C_11_H_14_ClFN_2_ respectively	NEK2	Inhibits NEK2	Pre-experimental (in silico)	No off-targets have been described	[207]
(5*Z*)-2-hydroxy-4-methyl-6-oxo-5-[(5-phenylfuran-2-yl)methylidene]-5,6-dihydropyridine-3-carbonitrile) or compound 8	C_22_H_24_F_3_N_8_O_2_	NEK6 NEK1	Inhibits NEK6. Displays anti-proliferative effects on several cancer cell lines with low IC50 values. Induces cell cycle arrest in the G_2_/M phases and has a synergistic effect with cisplatin and paclitaxel in ovarian cancer. It can inhibit NEK1.	Experimental	Inhibits NEK6 and NEK1. No off-targets have been described	[208]
Metformin	C_4_H_11_N_5_	NEK7	Metformin inhibits NEK7 expression in an experimental diabetic periodontitis model	Approved for type II diabetes	Activates AMPK, inhibits electron transfer flavoprotein-ubiquinone oxidoreductase (ETFDH), and glycerol-3-phosphate dehydrogenase [NAD(+)] (GPD1)	[175,209,210,211]
Ethyl 1-(2-hydroxypentyl) 5-(3-(3-(trifluoromethyl) phenyl)ureido)-1*H*-pyrazole-4-carboxylate or GeGe3	-	NEK10	Screening for GeGe3-targeted kinases revealed NEK10 as candidate targets, such as Aurora B. Inhibits physiological and tumor angiogenesis.	Experimental	Strongly inhibits aurora B, aurora C, NEK 10, polo like kinases 2 and 3 (PLK2/PLK3), dystrophia myotonica protein kinase 1 (DMPK), and calcium/calmodulin-dependent protein kinase type 1 (CaMK1)	[212]
Dabrafenib	C_23_H_2_0F_3_N_5_O_2_S_2_	NEK11	Reduces the proliferation and regression of tumors in xenograft models.	Approved for the treatment of metastatic melanoma with BRAF V600E mutations	Inhibits serine/threonine-protein kinase B-raf (BRAF1), proto-oncogene c-RAF (RAF), serine/threonine-protein kinase SIK1 (SIK-1), NEK11, and LIM domain kinase 1 (LIMK-1)	[213,214]
